# Prognostic Value of Bioactive Adrenomedullin in Critically Ill Patients with COVID-19 in Germany: An Observational Cohort Study

**DOI:** 10.3390/jcm10081667

**Published:** 2021-04-13

**Authors:** Tim-Philipp Simon, Christian Stoppe, Thomas Breuer, Lara Stiehler, Michael Dreher, Alexander Kersten, Stefan Kluge, Mahir Karakas, Elisabeth Zechendorf, Gernot Marx, Lukas Martin

**Affiliations:** 1 Department of Intensive and Intermediate Care, University Hospital RWTH Aachen, 52074 Aachen, Germany; tsimon@ukaachen.de (T.-P.S.); cstoppe@ukaachen.de (C.S.); tbreuer@ukaachen.de (T.B.); lara.stiehler@rwth-aachen.de (L.S.); ezechendorf@ukaachen.de (E.Z.); gmarx@ukaachen.de (G.M.); 2 Department of Pneumology and Intensive Care Medicine, University Hospital RWTH Aachen, 52074 Aachen, Germany; mdreher@ukaachen.de (M.D.); akersten@ukaachen.de (A.K.); 3 Department of Intensive Care Medicine, University Medical Center Hamburg-Eppendorf, 20251 Hamburg, Germany; skluge@uke.de (S.K.); m.karakas@uke.de (M.K.)

**Keywords:** ARDS, endothelial dysfunction, bioactive adrenomedullin, biomarker, COVID-19, ECMO

## Abstract

The coronavirus disease 2019 (COVID-19) pandemic has placed a significant burden on hospitals worldwide. Objective biomarkers for early risk stratification and clinical management are still lacking. The aim of this work was to determine whether bioactive adrenomedullin can assist in the risk stratification and clinical management of critically ill COVID-19 patients. Fifty-three patients with confirmed COVID-19 were included in this prospective observational cohort study between March and April 2020. Bioactive adrenomedullin (bio-ADM) plasma concentration was measured daily for seven days after admission. The prognostic value and clinical significance of bio-ADM plasma levels were evaluated for the severity of respiratory failure, the need for extracorporeal organ support and outcome (28-day mortality). Bio-ADM levels increased with the severity of acute respiratory distress syndrome (ARDS; *p* < 0.001) and were significantly elevated in invasively ventilated patients (*p* = 0.006) and patients in need of extracorporeal membrane oxygenation (*p* = 0.040) or renal replacement therapy (RRT; *p* < 0.001) compared to patients without these conditions. Non-survivors showed significantly higher bio-ADM levels than survivors (*p* = 0.010). Bio-ADM levels predicted 28-day mortality (C-index 0.72, 95% confidence interval 0.56–0.87, *p* < 0.001). Bio-ADM plasma levels correlate with disease severity, the need for extracorporeal organ assistance, and outcome, and highlight the promising value of bio-ADM in the early risk stratification and management of patients with COVID-19.

## 1. Introduction

As of the 18 May 2020, the worldwide COVID-19 pandemic had caused over 300,000 global deaths, with approximately 4.5 million confirmed cases [1]. Even in countries with high intensive care unit (ICU) capacity, such as Germany, the medical, societal, and economic impacts of the COVID-19 pandemic reveal unseen effects on the overall population mortality [2]. In Germany, particularly in the federal state of North Rhine-Westphalia as early as February 2020, a large number of individuals were infected and subsequently became ill. Consequently, the University Hospital Rheinisch-Westfälische Technische Hochschule (RWTH) Aachen became one of the academic centers treating severely ill COVID-19 very early on [3]. Infection with severe acute respiratory syndrome-related coronavirus 2 (SARS-CoV-2) is mainly characterized by fever, pneumonia, lymphopenia and exhausted lymphocytes, which may ultimately lead to the development of endothelial dysfunction and organ failure, necessitating a prolonged ICU stay with the need for organ support [4].

Although the evidence is continuously growing that the commonly used but rather nonspecific interleukin (IL)-6, C-reactive protein (CRP) and D-dimer levels are significantly elevated and linked to poor outcomes in most cases, robust biomarkers for the early risk stratification and clinical management of COVID-19 patients are still lacking, however, they are urgently needed [5,6,7]. Establishing objective biomarkers could ensure the efficient allocation of medical resources and inform physician decision-making to assign beds, ventilators, renal replacement therapy (RRT), and extracorporeal membrane oxygenation (ECMO) therapy while addressing the challenge of the best allocation of limited medical resources [8].

Respiratory and cardiovascular disorders are rapidly emerging as key threats in the disease development of COVID-19 [9]. The impact of these pathologies is of particular clinical relevance, as the lung is one major organ with a high proportion of endothelial cells. In fact, adrenomedullin (ADM) has been shown to play a key role in regulating vascular (hyper) permeability and endothelial stability/integrity in patients with severe infection [10] and has recently been presumed to be associated with COVID-19-induced endotheliitis [11]. ADM is ubiquitously expressed in human tissues. It is mainly expressed in endothelial and vascular smooth muscle cells [12,13], and to a lesser extent also in other tissues such as the adrenal medulla, intestines, heart, aortic skeletal muscle, kidneys, and the lungs [14,15]. The impairment of vascular integrity and an increase in endothelial permeability, marked by increased ADM plasma levels, are triggered by proinflammatory cytokines and the degradation of the basement membrane by matrix metalloproteinases that are released by activated endothelial cells. Endothelial cell infection, inflammation and activation with the subsequent interruption of endothelial barrier function has been described in COVID-19 patients [16,17]. In fact, Ackerman et al. described histopathological findings in the lungs of a small group of patients who died after developing COVID-19 disease. He and his coworkers found multifocal endotheliitis, endothelial injury and angiogenesis compared to patients who died after influenza infection. Thus, there is solid evidence that indicates an important role of the endothelium in the pathogenesis of COVID-19 [18].

Due to the different biological backgrounds (both ADM and mid-regional MR-proADM are derived from the same precursor (pro-ADM)), their stoichiometric relationship is imperfect, as ADM requires a C-terminal amidation to become biologically active adrenomedullin (bio-ADM) [19]. Bio-ADM has also been described as a therapeutic target to treat patients with septic shock. The humanized monoclonal antibody, Adrecizumab, aims to improve endothelial function and was recently investigated in a phase II clinical trial [20]. Adrecizumab was used for compassionate use in an uncontrolled study of eight severe COVID-19 patients, showing a possible beneficial effect on the outcome [21]. Supported by these results and the clinical experience with bio-ADM, we started to measure bio-ADM in our routine laboratory, and not only in COVID-19 patients. Other derivatives of the ADM precursor are likely to have a closer association with the patient’s endothelial status.

Previous studies in critically ill patients identified that bio-ADM, a marker for endothelial dysfunction [22], correlates with severe complications such as severe hypotension, edema formation, ionotropic/vasopressor use, need for organ support and subsequent organ failure [23,24]. Considering that endothelial dysfunction and pulmonary edema are of paramount relevance in the pathophysiology and ultimately in the clinical course of acute respiratory distress syndrome (ARDS) [25], we hypothesized that high and/or rising bio-ADM levels might predict a severe course of COVID-19 infection with the possible need for extracorporeal organ support. To evaluate whether bio-ADM plasma levels can assist in the clinical decision-making process for the adequate treatment of COVID-19 patients, we implemented daily measurements of bio-ADM in our ICU to identify patients with severe ARDS, the need for organ support, such as invasive ventilation, ECMO, and RRT, and those at risk of short-term death.

## 2. Materials and Methods

### 2.1. Study Population and Data Collection

After ethical approval (Ethical Committee of RWTH University, EK 100/20), this prospective observational study was performed between the 13 March and 16 April 2020 at the University Hospital RWTH Aachen, Germany. All patients or their legal representatives provided written informed consent. All patients with positive SARS-CoV-2 PCR results and ICU admission were included in this study. The exclusion criteria were age < 18 years old, pregnancy, and palliative care. ARDS was defined according to the Berlin definition [26] and validated by a blinded physician and expert in respiratory/critical care medicine who was independent of the study group and only had access to the data relevant for the classification. The analysis was carried out using real-time reverse transcription PCR (RT-PCR). The treatment of patients followed the standards of care in our ICU, including mechanical ventilation, veno-venous ECMO and RRT, if needed. The decision on the use of veno-venous ECMO therapy was based on the recently published Extracorporeal Life Support Organization (ELSO) consensus guidelines [27]. All parameters, including demographics, vital signs, laboratory values, blood gas analyses and organ support, were extracted from the patient data management system (Intellispace Critical Care and Anesthesia (ICCA) system, Philips, The Netherlands).

### 2.2. Bio-ADM Measurement

Blood was sampled on the day of admission and on a daily basis until day 7 for the analysis of bio-ADM and standard laboratory parameters. Bio-ADM was measured in EDTA plasma with a one-step luminescence sandwich immunoassay (SphingoTec GmbH, Hennigsdorf, Germany) [19]. In brief, 100 µL samples were incubated under agitation for one hour at room temperature with 150 µL of detection antibody directed against the N-terminus of bio-ADM in a microtiter plate coated with monoclonal antibody directed against mid-regional bio-ADM. Synthetic human bio-ADM was used as the calibrator. After washing, the chemiluminescence signal was measured in a microtiter plate luminescence reader (Centro LB960, Berthold Technologies, Bad Wildbad, Germany). The assay had a lower detection limit of 3 pg/mL. In a reference population of 200 healthy individuals, the median (99 percentile) bio-ADM level was 20.7 pg/mL (43 pg/mL) [28]. The bio-ADM cutoff value of 70 mg/dL was previously reported for ICU settings by Marino et al. [28] and was subsequently applied in the work of Mebazaa et al. [23] and Blet et al. [29].

### 2.3. Statistics

Values are expressed as the median and interquartile range (IQR), or count and percentage, as appropriate. Group comparisons of continuous variables were performed using the Kruskal–Wallis test. Categorical data were compared using Pearson’s chi-squared test for count data. Biomarker data were log-transformed. Boxplots were used to illustrate the differences in bio-ADM in categorical variables. Cox proportional hazards regression modeling was used to analyze the effect of (log-transformed) bio-ADM on survival in univariable analyses. The assumption of proportional hazards was tested. The predictive value of a model was assessed by the model likelihood ratio chi-square statistic. The concordance index (C index) is given as an effect measure. It is equivalent to the concept of the area under the curve (AUC) adopted for a binary outcome. The Kaplan–Meier survival curves were plotted and used for illustrative purposes. All statistical tests were 2-tailed, and a two-sided *p*-value of 0.05 was considered significant.

## 3. Results

In this cohort study, 53 patients with COVID-19 were consecutively included after confirmed SARS-CoV-2 infection and the need for ICU admission (*n* = 40 male (76%), median (IQR) age 62 {57–70} years) (Table 1). The median ICU length of stay was 16 (7.5–20) days. Thirty-two patients (60%) were discharged from the ICU to the normal ward prior to day 28, eight patients (15%) remained in the ICU, and 13 patients (25%) died. Markers of systemic inflammation are shown in Table 1.

**Figure 1:**
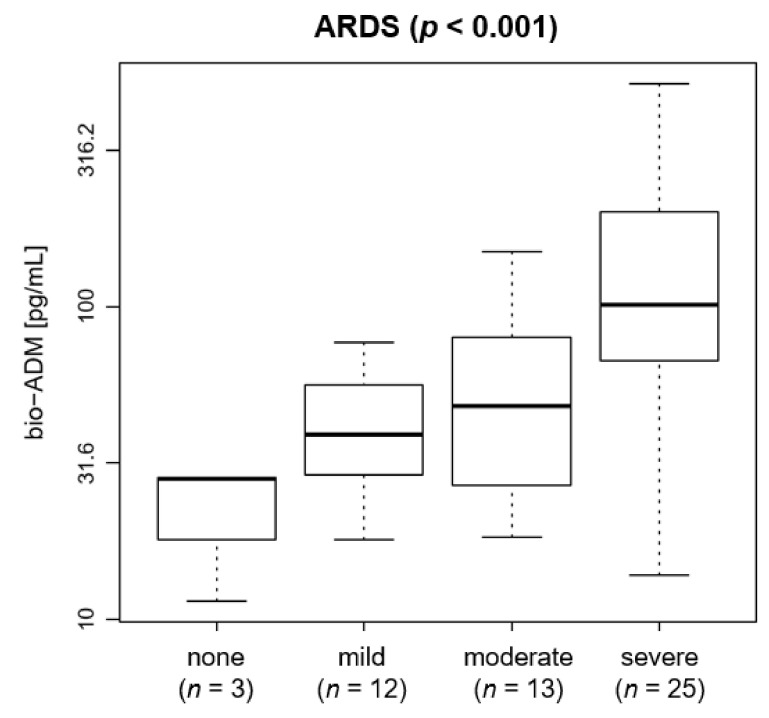
Boxplot of bio-ADM by ARDS in 53 COVID-19 patients at ICU admission (*p* < 0.001). The distribution of bio-ADM is shown as boxes, with the lower end of the boxes representing the 25th and the upper end of the boxes representing the 75th percentile (interquartile range), the middle line representing the median, and the whiskers showing the minimal and maximal bio-ADM levels. Horizontal line at 70 pg/mL. The y axis uses a logarithmic scale. ARDS: acute respiratory distress syndrome; bio-ADM: bioactive adrenomedullin; ICU: intensive care unit.

A high proportion of patients 72% (*n* = 38) presented with moderate or severe ARDS (25% moderate, 47% severe). Bio-ADM levels increased with the severity of ARDS (*p* < 0.001): bio-ADM increased from a median (IQR) of 28.3 {19.9–28.4} pg/mL for patients without ARDS to 39.0 {29.2–54.5} pg/mL for patients with mild ARDS, to 48.1 {26.9–79.8} pg/mL for patients with moderate ARDS and to 101.9 {67.0–201.1} pg/mL for patients with severe ARDS (Figure 1).

**Figure 2:**
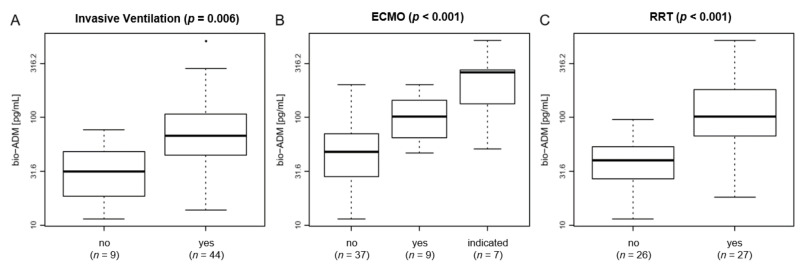
Boxplot of bio-ADM levels by (**A**) invasive ventilation (*p* = 0.006); (**B**) ECMO (*p* < 0.001); and (**C**) RRT (*p* < 0.001) in 53 COVID-19 patients upon ICU admission. The distributions of bio-ADM are shown as boxes, with the lower end of the boxes representing the 25th and the upper end of the boxes representing the 75th percentile (interquartile range), the middle line representing the median, and the whiskers showing the minimal and maximal bio-ADM levels. Outliers are shown as circles plotted beyond the whiskers. Patients who fulfilled the criteria for ECMO therapy but did not receive ECMO treatment were termed “indicated”. The y axis uses a logarithmic scale. bio-ADM: bioactive adrenomedullin; ECMO: extracorporeal membrane oxygenation; ICU: intensive care unit; RRT: renal replacement therapy.

The majority of patients (*n* = 44) received invasive ventilation during the ICU stay (Table 1). Bio-ADM levels were significantly increased in invasively ventilated patients compared to spontaneously breathing patients (68.2 {45.5–106.6} pg/mL vs. 31.8 {18.6–48.4} pg/mL, *p* = 0.006) (Figure 2A). Of note, bio-ADM levels on ICU admission were similarly elevated in patients who received invasive ventilation upon ICU admission (*n* = 38) compared to those patients who required mechanical ventilation in due course during the study period (*n* = 6) (69.8 {44.1–107.3} pg/mL vs. 63.2 {51.0–88.7} pg/mL).

Increased bio-ADM levels were observed in patients treated with veno-venous ECMO (*n* = 9) compared to patients without ECMO therapy (101.9 {65.0–144.1} pg/mL vs. 53.3 {29.2–91.0} pg/mL, *p* = 0.040) (Figure 2B). Notably, the highest bio-ADM levels were observed in patients who were eligible for ECMO therapy because of the severity of respiratory failure according to the ELSO consensus guideline (16) but were not treated with ECMO because of individual patient decree (262.1 {136.1–274.6} pg/mL, *p* < 0.001) (Figure 2B). Moreover, bio-ADM levels significantly correlated with the dose of norepinephrine (*r* = 0.47, *p* < 0.001).

With respect to kidney function, there was a notable correlation between bio-ADM and serum creatinine (*r* = 0.62, *p* < 0.001) on the day of admission. In line, significantly higher bio-ADM levels were found in patients receiving RRT compared to patients without RRT (101.9 {67.7–182.9} pg/mL vs. 40.2 {27.2–53.5} pg/mL, *p* < 0.001) (Figure 2C).

**Figure 3:**
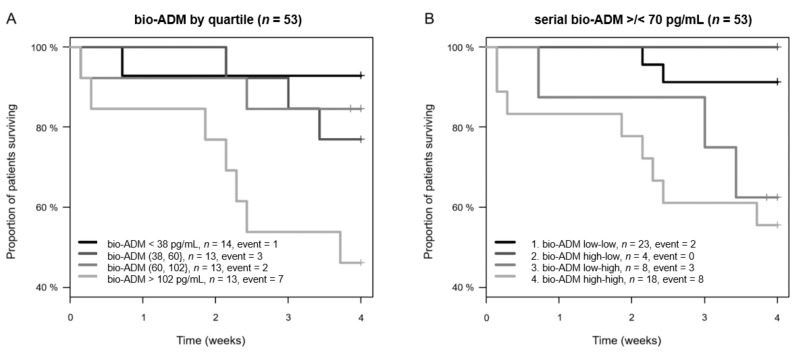
Kaplan–Meier plot for 28-day mortality for bio-ADM. Curves are plotted by (**A**) bio-ADM quartiles (for continuous bio-ADM) and (**B**) to illustrate the potential value of serial measurements of bio-ADM, by > or <70 pg/mL at admission and 48 h (*p* = 0.122). Patients with missing bio-ADM data at 48 h remain in their initial category. bio-ADM: bioactive adrenomedullin.

Bio-ADM levels on the day of admission were higher in non-survivors than in survivors (107.6 {51.0–262.1} pg/mL vs. 53.3 {29.2–91.0} pg/mL, *p* = 0.010). Notably, bio-ADM on the day of admission predicted 28-day mortality (C-index 0.72, 95% confidence interval (CI) 0.56–0.87, *p* < 0.001) (Figure 3A). Bio-ADM was independent and superior to laboratory biomarkers measured at the same time, including CRP (standardized HR 3.5 (95% CI 1.6–7.5), C index 0.50, *p* = 0.801); the added value of bio-ADM on top of CRP (*p* = 0.006), PCT (C index 0.67, *p* = 0.081; added value of bio-ADM *p* = 0.008), lactate (C index 0.53, *p* = 0.557; added value of bio-ADM *p* = 0.002), IL-6 (C index 0.54, *p* = 0.526; added value of bio-ADM *p* = 0.004) and creatinine (C index 0.68, *p* = 0.020; added value of bio-ADM *p* = 0.028).

We then elucidated the additional value of the serial measurement of bio-ADM for the prediction of 28-day mortality. Based on previous studies (18–22), we applied a cutoff value for bio-ADM of 70 pg/mL and grouped the patients accordingly (Figure 3B).

## 4. Discussion

The current COVID-19 pandemic is a serious challenge to hospitals worldwide, necessitating decision-making on limited critical care resources while the knowledge of the pathophysiology of this new disease is constantly evolving. Although most COVID-19 patients show mild to moderate symptoms, some develop multiple organ failure, including ARDS [30]. In the absence of specific treatment strategies, there is an urgent call for clear guidance by early risk-stratifying biomarkers to identify and to manage the increasing numbers of vulnerable COVID-19 patients in need of escalated intensive care treatment [31]. In the present study, the first German observational study in critically ill COVID-19 patients, we investigated bio-ADM as an early biomarker in 53 critically ill COVID-19 patients admitted to the ICU at the University Hospital RWTH Aachen. Increased bio-ADM levels at ICU admission were associated with the severity of ARDS, the subsequent need for organ support (mechanical ventilation, veno-venous ECMO, renal replacement therapy, and vasopressors) and 28-day mortality. Thus, our findings clearly highlight the potential of bio-ADM as a promising biomarker for the early risk stratification of critically ill patients suffering from COVID-19.

The cardiovascular and respiratory systems are substantially involved in the disease progression of COVID-19, as all cases showed pneumonia upon admission and relatively high rates of pulmonary and cardiovascular complications. ADM controls vasodilation and endothelial integrity. Endothelial function and barrier stability are developed and maintained by ADM, as confirmed by knockout animal models in which the underlying signaling pathways were abolished and edema formation and vascular leakages were observed [32]. Translating these findings into clinical practice, studies have shown that bio-ADM plasma levels correlate with multiple organ injuries, severe hypotension, edema formation, disease severity and mortality in patients with sepsis, while low and decreasing bio-ADM blood levels indicate improved outcomes [24,28,33]. Similar evidence exists for other indications where endothelial dysfunction is of relevance, such as in acute heart failure [34] and cardiogenic shock [35].

SARS-CoV-2 enters host cells via the angiotensin converting enzyme 2 receptor, which is expressed in the kidney, lung and heart [36]. Histological investigations of tissues from COVID-19 patients who suffered severe respiratory failure, needed organ support and even some who eventually died of multiple organ failure, revealed a substantial pathological alteration in the endothelial glycocalyx. The latter indicates that the endothelial dysfunction and disturbance of endothelial integrity are crucial mechanisms in the pathophysiology of COVID-19 [16,37]. The present findings clearly show an association between the bio-ADM levels at ICU admission and the severity of ARDS in patients suffering from COVID-19, indicating a central role of bio-ADM in the pathology of COVID-19-induced pulmonary injury. In addition, our data highlight the potential of bio-ADM to identify patients in need of invasive ventilation during the ICU stay, as patients with invasive ventilation revealed significantly increased levels of bio-ADM at ICU admission. These findings confirm and extend previous observational studies in septic patients, indicating an association between increased levels of bio-ADM and the need for mechanical ventilation [23]. Furthermore, bio-ADM plasma levels were closely related to the need for veno-venous ECMO therapy and RRT. While acknowledging that our findings do not establish a causal relationship between elevated bio-ADM plasma levels and endothelial dysfunction and the clinical course of COVID-19 patients, our data strengthen the importance of bio-ADM—and therefore endothelial dysfunction—playing a crucial role in the development of organ failure in critically ill COVID-19 patients [38]. However, it is important to mention that although our data indicate a strong predictive value of bio-ADM in regard to the clinical course of the patient, the causative relationship remains unclear and needs further investigation.

In this context, previous exploratory studies have already demonstrated significantly elevated cytokine levels, which may contribute to the development of shock and tissue damage in the heart, liver and kidney as well as respiratory failure in COVID-19 patients [39]. Although markers for organ functions, such as the heart and kidney, are regularly assessed in clinical practice, the endothelium, as one of the most relevant organs, is not routinely monitored, even if highly relevant for the homeostasis of organ function [40].

Several publications from China addressed the prognostic value of different parameters in predicting disease severity and short-term survival in COVID-19 patients [41,42,43,44,45,46]. Of note, although limited by a small size and the single center character, our data show significantly elevated bio-ADM levels in non-survivors compared to survivors with a moderate predictive value for 28-day mortality. These findings are in line with earlier work showing that bio-ADM levels correlate with disease severity and mortality in patients with sepsis [28,33].

## 5. Conclusions

In conclusion, this first German study on bio-ADM in critically ill COVID-19 patients shows the promising value of circulating bio-ADM in the early risk stratification of severe COVID-19 patients. Our work opens up for future randomized trials that prospectively evaluate bio-ADM as a new objective tool for the risk stratification and monitoring of patients suffering from COVID-19.

## Figures and Tables

**Table 1:** The baseline characteristics of 53 critically ill COVID-19 patients stratified by the severity of ARDS. The variables in the outcome section of Table 1, intubation and status on day 28, have three categories each, and the *p*-value compares all three.

Variable	All (*n* = 53)	None (*n* = 3)	Mild (*n* = 12)	Moderate (*n* = 13)	Severe (*n* = 25)	*p*-Value
Age (years, median (IQR))	62 {57–70}	53 {49–65}	61 {59–64}	62 {54–67}	66 {58–72}	0.767
Gender male, *n* (%)	40 (75.5)	3 (100)	10 (83.3)	6 (46.2)	21 (84.0)	0.039
Body mass index (kg/m^2^, median (IQR))	29.3 {24.9–32.6}	24.9 {24.7–28.2}	29.2 {26.3–34.9}	30.5 {6.7–35.2}	29.3 {24.7–31.3}	0.758
Temperature, max (°C, median (IQR))	38.1 {37.4–38.5}	38.1 {37.8–38.8}	38.1 {37.8–38.6}	38.2 {37.0–38.5}	38.0 {37.3–38.5}	0.934
Heart rate (bpm, median (IQR))	106 {89–114}	93 {86–107}	105 {93–109}	91 {72–103}	112 {104–121}	0.014
Respiratory rate (bpm, median (IQR))	25 {23–28}	24 {22–25}	24 {23–26}	25 {22–28}	25 {23–29}	0.678
SOFA score at the day of enrollment (points, median (IQR))	9.0 {7.0–11.0}	8.5 {7.8–9.3}	7.0 {6.0–9.5}	8.5 {7.8–10.0}	11.0 {9.0–11.0}	0.037
Blood gas analysis (at the day of enrollment)						
Arterial pH (median (IQR))	7.36 {7.30–7.42}	7.47 {7.38–7.49}	7.40 {7.37–7.44}	7.38 {7.33–7.43}	7.32 {7.28–7.36}	0.011
pCO_2_ (mmHg, median (IQR))	45.1 {39.3–52.0}	48.0 {42.1–71.3}	36.7 {33.8–41.2}	45.5 {43.2–52.0}	48.2 {42.1–55.4}	0.001
pO_2_ (mmHg, median (IQR))	79 {70–91}	71 {64–80}	92 {75–105}	79 {70–92}	79 {70–84}	0.345
SpO_2_ (%, median (IQR))	95 {94–98}	94 {93–94}	98 {96–99}	98 {95–100}	94 {93–97}	0.031
Horowitz index (mmHg/%, median (IQR))	114 {88–151}	133 {89–276}	224 {168–276}	115 {100–150}	94 {71–115}	0.002
Biomarker (at the day of enrollment, unless otherwise stated)						
bio-ADM (pg/mL, median (IQR))	59.9 {37.9–101.9}	28.3 {19.9–28.4}	39.0 {29.2–54.5}	48.1 {26.9–79.8}	101.9 {67.0–201.1}	<0.001
bio-ADM > 70 pg/mL, n (%)	22 (41.5)	0 (0)	1 (8.3)	4 (30.8)	17 (68.0)	0.002
Lactate (mmol/L, median (IQR))	1.0 {0.8–1.4}	0.7 {0.5–1.0}	0.8 {0.7–0.9}	0.9 {0.7–1.5}	1.3 {1–1.7}	0.003
IL-6 (pg/mL, median (IQR))	158.4 {97.4–337.4}	51.9 {34.5–69.4}	65.7 {46.9–93.5}	211.2 {141.3–519.9}	251.5 {151.2–475.2}	0.001
PCT (ng/mL, median (IQR))	0.53 {0.13–1.89}	0.07 {0.06–0.08}	0.14 {0.11–0.25}	0.22 {0.11–0.69}	1.46 {0.66–5.06}	<0.001
CRP (nmol/L, median (IQR))	175 {117–326}	182 {182–182}	80 {34–142}	256 {124–298}	251 {158–350}	0.002
WBC (10^3^/mm^3^, median (IQR))	9.3 {6.6–13.0}	10.4 {9.3–11.9}	6.2 {5.7–10.8}	8.0 {7.4–9.4}	10.1 {8.0–13.9}	0.120
Platelets (10^3^/µL, median (IQR))	228 {198–329}	202 {200–292}	197 {140–236}	237 {204–328}	263 {204–338}	0.242
Creatinine (mg/dL, median (IQR))	1.1 {0.8–2.2}	0.7 {0.6–0.7}	1.0 {0.8–1.2}	0.9 {0.6–1.1}	1.8 {1.2–3.0}	0.004
Comorbidities						
Arterial hypertension, *n* (%)	27 (50.9)	1 (33.3)	5 (41.7)	9 (69.2)	12 (48.0)	0.455
Diabetes mellitus, *n* (%)	13 (24.5)	0 (0)	1 (8.3)	3 (23.1)	9 (36.0)	0.215
Ischemic heart disease, *n* (%)	10 (18.9)	0 (0)	2 (16.7)	4 (30.8)	4 (16.0)	0.557
Embolism/thrombosis, *n* (%)	6 (11.3)	1 (33.3)	1 (8.3)	3 (23.1)	1 (4.0)	0.197
Cardiac arrhythmia, *n* (%)	6 (11.3)	0 (0)	1 (8.3)	0 (0)	5 (20.0)	0.259
Cerebral vascular disease, *n* (%)	5 (9.4)	0 (0)	2 (16.7)	0 (0)	3 (12.0)	0.459
COPD, *n* (%)	6 (11.3)	1 (33.3)	2 (16.7)	1 (7.7)	2 (8.0)	0.525
Other lung diseases, *n* (%)	2 (3.8)	1 (33.3)	1 (8.3)	0 (0)	0 (0)	0.025
Chronic kidney disease, *n* (%)	8 (15.1)	0 (0)	2 (16.7)	3 (23.1)	3 (12.0)	0.708
Tumor disease, *n* (%)	4 (7.5)	0 (0)	3 (25.0)	1 (7.7)	0 (0)	0.057
Smoker, *n* (%)	3 (5.7)	0 (0)	1 (8.3)	2 (15.4)	0 (0)	0.247
Treatment in the ICU (first 14 days, unless otherwise stated)						
ICU length of stay (days, median (IQR))	16 {7.5–20.0}	6 {4.0–9.5}	7.5 {3.0–10.5}	19.5 {16.5–23.0}	17.5 {15.0–21.0}	0.004
Highest dose of Norepinephrine during the first 7 days (µg/kg/min, median (IQR))	0.15 {0.06–0.29}	0.07 {0.03–0.11}	0 {0–0.09}	0.15 {0.06–0.18}	0.29 {0.13–0.35}	<0.001
Anticoagulation, *n* (%)	15 (28.3)	1 (33.3)	2 (16.7)	3 (23.1)	9 (36.0)	0.627
Antiplatelet, *n* (%)	15 (28.3)	0 (0)	4 (33.3)	6 (46.2)	5 (20.0)	0.238
Antihypertensive, *n* (%)	32 (60.4)	1 (33.3)	8 (66.7)	10 (76.9)	13 (52.0)	0.343
Immunosuppressant, *n* (%)	9 (17)	1 (33.3)	2 (16.7)	4 (30.8)	2 (8.0)	0.289
Analgesics, *n* (%)	8 (15.1)	1 (33.3)	4 (33.3)	1 (7.7)	2 (8.0)	0.143
Ventilation						
Intubation during ICU stay, *n* (%)	44 (83.0)	1 (33.3)	5 (41.7)	13 (100)	25 (100)	0.006
Intubation:						
never, *n* (%)	9 (17.0)	2 (67.7)	7 (58.3)	0 (0)	0 (0)	
at admission, *n* (%)	38 (71.7)	1 (33.3)	3 (25.0)	12 (92.3)	22 (88.0)	0.022
later, n (%)	6 (11.3)	0 (0)	2 (16.7)	1 (7.7)	3 (12.0)	
Outcome						
Death 28 days, *n* (%)	13 (24.5)	1 (33.3)	0 (0)	1 (7.7)	11 (44.0)	0.011
Status on day 28:						0.001
discharged, *n* (%)	32 (60.4)	2 (66.7)	12 (100)	11 (84.6)	7 (28.0)	
in ICU post day 28, *n* (%)	8 (15.1)	0 (0)	0 (0)	1 (7.7)	7 (28.0)	
death 28 days, *n* (%)	13 (24.5)	1 (33.3)	0 (0)	1 (7.7)	11 (44.0)	

Variables are given as the median (interquartile range) or number (%). ARDS, acute respiratory distress syndrome; bio-ADM, bioactive adrenomedullin; COPD, chronic obstructive pulmonary disease; CRP, C-reactive protein; ECMO, extracorporeal membrane oxygenation; FiO2, fraction of inspired oxygen; ICU, intensive care unit; IL-6, interleukin-6; pCO2, partial pressure of carbon dioxide; PCT, procalcitonin; PEEP, positive end-expiratory pressure; pO2, partial pressure of oxygen; RRT, renal replacement therapy; SpO2, peripheral capillary oxygen saturation; SOFA, sequential organ failure assessment; WBC, white blood cell counts.

## Data Availability

The raw data supporting the conclusions of this article will be made available by the authors, without undue reservation.
